# Distinct radiation responses after *in vitro* mtDNA depletion are potentially related to oxidative stress

**DOI:** 10.1371/journal.pone.0182508

**Published:** 2017-08-03

**Authors:** Marike W. van Gisbergen, An M. Voets, Rianne Biemans, Roland F. Hoffmann, Marie-José Drittij-Reijnders, Guido R. M. M. Haenen, Irene H. Heijink, Kasper M. A. Rouschop, Ludwig J. Dubois, Philippe Lambin

**Affiliations:** 1 Department of Radiation Oncology (MaastRO Lab), GROW – School for Oncology and Developmental Biology, Maastricht University Medical Centre, Maastricht, The Netherlands; 2 Department of Clinical Genomics, GROW – School for Oncology and Developmental Biology, Maastricht University Medical Centre, Maastricht, The Netherlands; 3 Department of Pathology and Medical Biology, University of Groningen, University Medical Center Groningen, Groningen, The Netherlands; 4 Department of Toxicology, NUTRIM - School for Nutrition, Toxicology, and Metabolism, Maastricht University Medical Centre, Maastricht, The Netherlands; 5 Department of Pulmonology, University of Groningen, University Medical Center Groningen, Groningen, The Netherlands; 6 Groningen Research Institute for Asthma and COPD (GRIAC), University of Groningen, Groningen, The Netherlands; University of South Alabama Mitchell Cancer Institute, UNITED STATES

## Abstract

Several clinically used drugs are mitotoxic causing mitochondrial DNA (mtDNA) variations, and thereby influence cancer treatment response. We hypothesized that radiation responsiveness will be enhanced in cellular models with decreased mtDNA content, attributed to altered reactive oxygen species (ROS) production and antioxidant capacity. For this purpose BEAS-2B, A549, and 143B cell lines were depleted from their mtDNA (ρ^0^). Overall survival after irradiation was increased (p<0.001) for BEAS-2B ρ^0^ cells, while decreased for both tumor ρ^0^ lines (p<0.05). In agreement, increased residual DNA damage was observed after mtDNA depletion for A549 and 143B cells. Intrinsic radiosensitivity (surviving fraction at 2Gy) was not influenced. We investigated whether ROS levels, oxidative stress and/or antioxidant responses were responsible for altered radiation responses. Baseline ROS formation was similar between BEAS-2B parental and ρ^0^ cells, while reduced in A549 and 143B ρ^0^ cells, compared to their parental counterparts. After irradiation, ROS levels significantly increased for all parental cell lines, while levels for ρ^0^ cells remained unchanged. In order to investigate the presence of oxidative stress upon irradiation reduced glutathione: oxidized glutathione (GSH:GSSG) ratios were determined. Irradiation reduced GSH:GSSG ratios for BEAS-2B parental and 143B ρ^0^, while for A549 this ratio remained equal. Additionally, changes in antioxidant responses were observed. Our results indicate that mtDNA depletion results in varying radiation responses potentially involving variations in cellular ROS and antioxidant defence mechanisms. We therefore suggest when mitotoxic drugs are combined with radiation, in particular at high dose per fraction, the effect of these drugs on mtDNA copy number should be explored.

## Introduction

Under normal physiological conditions, mitochondria are important organelles in the cell. One of their key functions is the production of adenosine triphosphate (ATP) via the oxidative phosphorylation system (OXPHOS), thereby providing the cell with its essential energy supply. It has been observed that tumor cells rely less on OXPHOS but are more dependent on aerobic glycolysis, also known as the Warburg effect [1]. New findings have revealed that this metabolic shift could be the result of metabolic reprogramming from a more OXPHOS to a more glycolytic phenotype (uncoupled glycolysis) regardless of oxygen presence in the tumor tissue, supporting the tumor’s anabolic growth and micro-environmental adaptations. This may be a possible explanation for an increased metastatic potential of tumor cells [2,3]. Potential causes for such metabolic reprogramming could be changes in the mitochondrial genome (mtDNA) such as deletions, substitutions and even mtDNA copy number variations, leading to dysfunctional mitochondria [4]. Mitochondria play an important role in processes like reactive oxygen production, redox signalling and apoptosis, while these processes influence significantly radiotherapy (RT) response [5,6,7,8]. Furthermore, repair of RT-induced damage is dependent on mitochondrial energy supply which heavily relies upon the functionality of mitochondria [9]. mtDNA alterations, such as mutations, deletions or copy number variations, may therefore influence RT response. These variations can be potentially caused by mitotoxic agents, such as cisplatin [10] or doxorubicin [11]. Cisplatin is able to induce a decrease in mtDNA copy number and impairment of mtRNA synthesis [10]. Doxorubicin intercalates with mtDNA and thereby contributes to mitochondrial toxicity [11]. Different types of alterations of the mtDNA genome have been found to be related to cancer and treatment outcome [4]. Cells fully depleted from their mitochondrial genome, Rho-0 (ρ^0^) cells, are an extreme *in vitro* model to investigate the association between radiation outcome and dysfunctional mitochondria due to abnormalities in the mitochondrial genome [12,13]. Several contradictory results on cellular mtDNA depletion using various (tumor) cell lines have been published. Different radiation responses were observed varying between a radioresistant phenotype to no difference in radiation response *in vitro* [14,15]. However, an increased radiation response was observed in an *in vivo* model [16]. To our knowledge no mechanistic insights have been proposed to explain the observed differences in radiation response of mtDNA depleted cell lines. Therefore, the aim of this study was to elicudate the mechanistic insights underlying the radiation response of mtDNA depleted cells. We hypothesized that reduced mitochondrial function after mtDNA depletion changes the radiation response and this is dependent on altered ATP production, ROS production and on the cells’ antioxidant capacity.

## Material and methods

### Cell culture model

The parental 143B and mtDNA depleted 143B Rho-0 (ρ^0^) osteosarcoma cells were cultured in Gibco’s Dulbecco’s modified Eagle’s medium (DMEM, D-glucose 4.5 g/l) with 10% fetal bovine serum (FBS; Lonza), the latter supplemented with 150 μg/ml uridine and 100 μg/ml bromodeoxyuridine (Sigma-Aldrich) [17]. A549 (alveolar type-II carcinoma cells) ρ^0^ cells were created by Prof. Dr. Ian Holt (Cambridge University, United Kingdom) and parental and ρ^0^ cells were kindly provided by Dr. Lodovica Vergani (Padova University, Italy). mtDNA depletion of BEAS-2B (adenovirus-12 SV40 hybrid virus transformed bronchial epithelial) cells was accomplished by culturing cells in medium supplemented with ethidium bromide (50 ng/ml; Sigma-Aldrich). Both A549 and BEAS-2B cells were cultured in DMEM (D-glucose 4.5 g/l) supplemented with 25% FBS, vitamins, 1X essential and non-essential amino acids (Sigma-Aldrich) and 50 μg/ml uridine (Acros Organics).

### mtDNA copy number determination

Confirmation of mtDNA depletion was obtained by performing quantitive PCR. DNA was isolated using the gentra puregene kit (Qiagen). Ratios for the nuclear DNA (nDNA) the B2M gene and mitochondrial DNA (mtDNA) D-Loop were obtained in order to determine the mtDNA content. Primer secquences can be found in S1 Table. Quantitative PCR was performed on the 7900HT Fast Real-Time PCR System (Applied Biosystems). The PCR mixture contained 5ng/μl DNA, 0.3 μM forward and reverse primer and 1x master-mix (SensiMix SYBR^®^ HiRox kit, Bioline Reagents). The cycling conditions were: 2′ 50°C, 10’ 95°C, 40 cycles of 15′′ at 95°C + 1′ 60°C.

### Proliferation and clonogenic survival assay

Proliferation was monitored during 7 days using the IncuCyte FLR after seeding 2500 cells/well. For clonogenic survival analysis, cells were seeded on day 0 and irradiated using a 225kV Philips X-ray tube on day 1. Subsequently, cells were trypsinized and plated in triplicate for clonogenic survival. Cells were allowed to form colonies during 10 days, fixed and stained with a 0.4% methylene blue (Sigma-Aldrich) in 70% ethanol solution. Colonies were defined as >50 cells [18].

### Metabolic profiling

Cells were seeded at an optimized cell density of 3x10^4^ cells/well (BEAS-2B) or 1.5x10^4^ cells/well (143B and A549). Metabolic profiles were generated by replacing the growth media for assay media 1 hour before using the Seahorse XF96 extracellular Flux analyzer (Seahorse Bioscience) according to manufacturer’s guidelines [19,20]. A mitochondrial stress test was established measuring the oxygen consumption rate (OCR) after subsequent injections of 1 μM oligomycin, optimized FCCP concentrations 0.3 μM (A549), 0.5 μM (143B) or 0.6 μM (BEAS-2B) and 1 μM mixture of rotenone and antimycin A (Sigma-Aldrich) and spare capacity, proton leak and ATP production were calculated according to the Seahorse Bioscience guidelines. The glycolysis stress test was performed by measuring the extracellular acidification rate (ECAR) after sequential addition of 10 mM glucose, optimized oligomycin concentration 1.0 μM (all cell lines) and 0.1 M 2-deoxyglucose (2-DG) (Sigma-Aldrich). Calculations of the glucose metabolism and glycolytic reserve were done according to the Seahorse Bioscience guidelines. Baseline OCR or ECAR was determined prior to the first compound injection using a mixing period of 5 minutes and a measurement period of 3 minutes followed by 3 loops of mixing and measuring for 3 minutes each. Every injection was followed by the same measurement protocol of a mixing period of 5 minutes and a measurement period of 3 minutes followed by 3 loops of mixing and measuring for 3 minutes.

### Molecular assays

ATP levels were measured based on the Cell-TiterGlo Luminescent cell viability test (Promega) on the Glomax 96 well luminometer (Promega). Levels of extracellular L-Lactic acid were measured by using the L-lactic acid kit (Biosentec) according to manufacturer’s guidelines. Both ATP and L-lactic acid levels were corrected for cell counts. Formation of reactive oxygen species (ROS) was detected 24 hours after ionizing radiation (4Gy). Cells were exposed for 1 hour to 20 μM dihydrorhodamine 123 (Invitrogen) and subsequently washed with PBS before trypsinization and cell straining in order to obtain a single cell suspension. The cell suspensions were exposed to 2 μg/ml final concentration of propidium iodide (Sigma-Aldrich) 1 minute before FACS analysis. Levels of ROS were determined in the propidium iodide negative population by flow cytometry (BD FACS Canto II) and expressed using mean fluorescent intensity [21]. Glutathione levels (GSH and GSSG) were measured in the cell lysates as described previously [22].

### *γ-H2AX* immunocytochemistry

Cells were fixed with 100% methanol at −20°C. Subsequently cells were permeabilized with 0.05% Tween-PBS and normal goat serum was used as blocking agent. Cells were stained with a primairy anti-phospho(Ser139)-H2AX antibody (1:500, Millipore) followed by anti-rabbit Alexa Fluor 488 (1:500, Invitrogen) as secundairy antibody. Hoechst 33342 (20 ug/ml, Sigma–Aldrich) was used as nuclear counter stain. Images were acquired using a Leica TCS SPE confocal microscope.

### RNA isolation and quantitative PCR analysis

mRNA was extracted using the NucleoSpin RNA II kit (Bioke) using iScript cDNA Synthesis Kit (BioRad). Both methods were performed according to the manufacturers’ instructions. Quantitative PCR was performed on the CFX96 (Biorad). The abundance of the genes of interest were detected with SYBR^®^ Green I (Eurogentec). Values for each gene were normalized to 18S expression levels. Primer sequences are listed in the S1 Table.

### Statistics

All statistical analyses were performed with GraphPad Prism version 5.03 for Windows (GraphPad Software, 2009, California, USA). A non-parametric Mann–Whitney *U* test for small groups was used to determine the statistical significance of differences between two independent groups of variables. Clonogenic survival curves were compared using an extra sum F-test. For all tests, a p<0.05 was considered significant.

## Results

**Fig 1:**
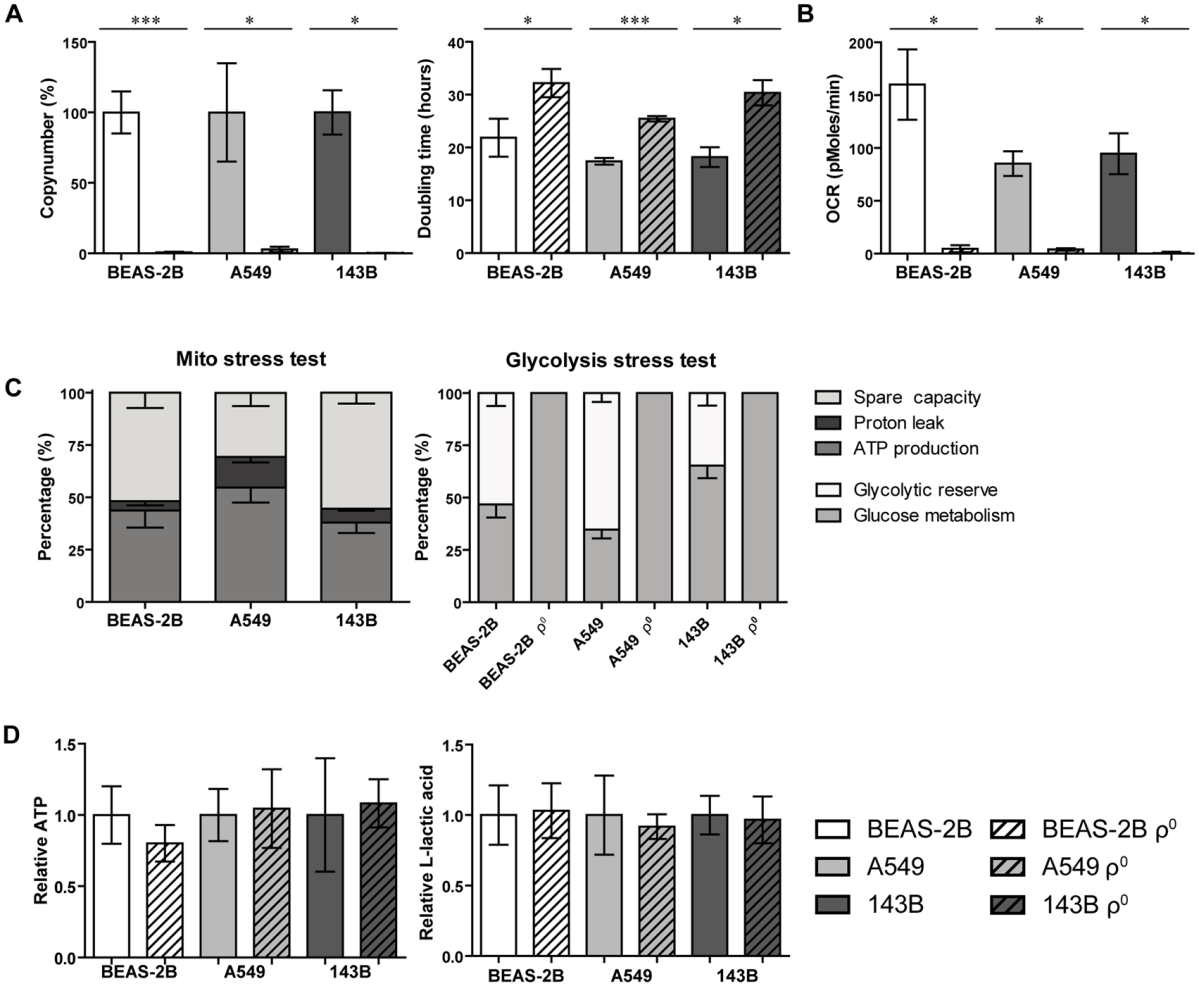
Validation of mtDNA depleted cell lines. **A.** qPCR assessed mtDNA copy number levels expressed in percentage normalized to each parental (left panel) and the doubling time for all investigated cell lines (right panel). **B.** Basal respiration expressed as oxygen consumption rate (OCR) in function of time (minutes). **C.** Stacked plot of mitochondrial (left panel) or glycolysis (right panel) stress test after measuring OCR or extracellular acidification rate (ECAR) respectively. **D.** Relative ATP levels in A.U. corrected for cell number (left panel) and L-Lactic acid levels in g/l corrected for cell number (right panel). Data represent the mean ± SEM from at least 3 independent biological experiments. * p<0.05, *** p<0.001.

To investigate if extreme changes in OXPHOS capacity affect radiosensitivity, we used ρ^0^ cell lines with different genetic backgrounds. All our ρ^0^ cell lines showed a strong and significant decrease in mtDNA copy number (Fig 1A; BEAS-2B p<0.001; A549 p<0.05; 143B p<0.05). Additionally, proliferation was decreased as evaluated by an increased doubling time (on average 54%) for all ρ^0^ cell lines (Fig 1A). We confirmed the drastic effect on OXPHOS upon mtDNA depletion as basal respiration was almost absent (p<0.05) in the BEAS-2B, A549 and 143B ρ^0^ (Fig 1B) while the parental cells had a functional OXPHOS system with spare capacity (Fig 1C). To compensate their loss of OXPHOS function, ρ^0^ cells showed an enhanced (p<0.05) glucose metabolism accompanied with a loss in glycolytic reserve (Fig 1C; S1 Fig). The mtDNA depletion did not result in reduced cellular ATP levels and no differences in lactic acid production were observed between ρ^0^ and their parental counterparts (Fig 1D). Together our results indicate that mtDNA depleted cells upscaled their glucose metabolism.

**Fig 2:**
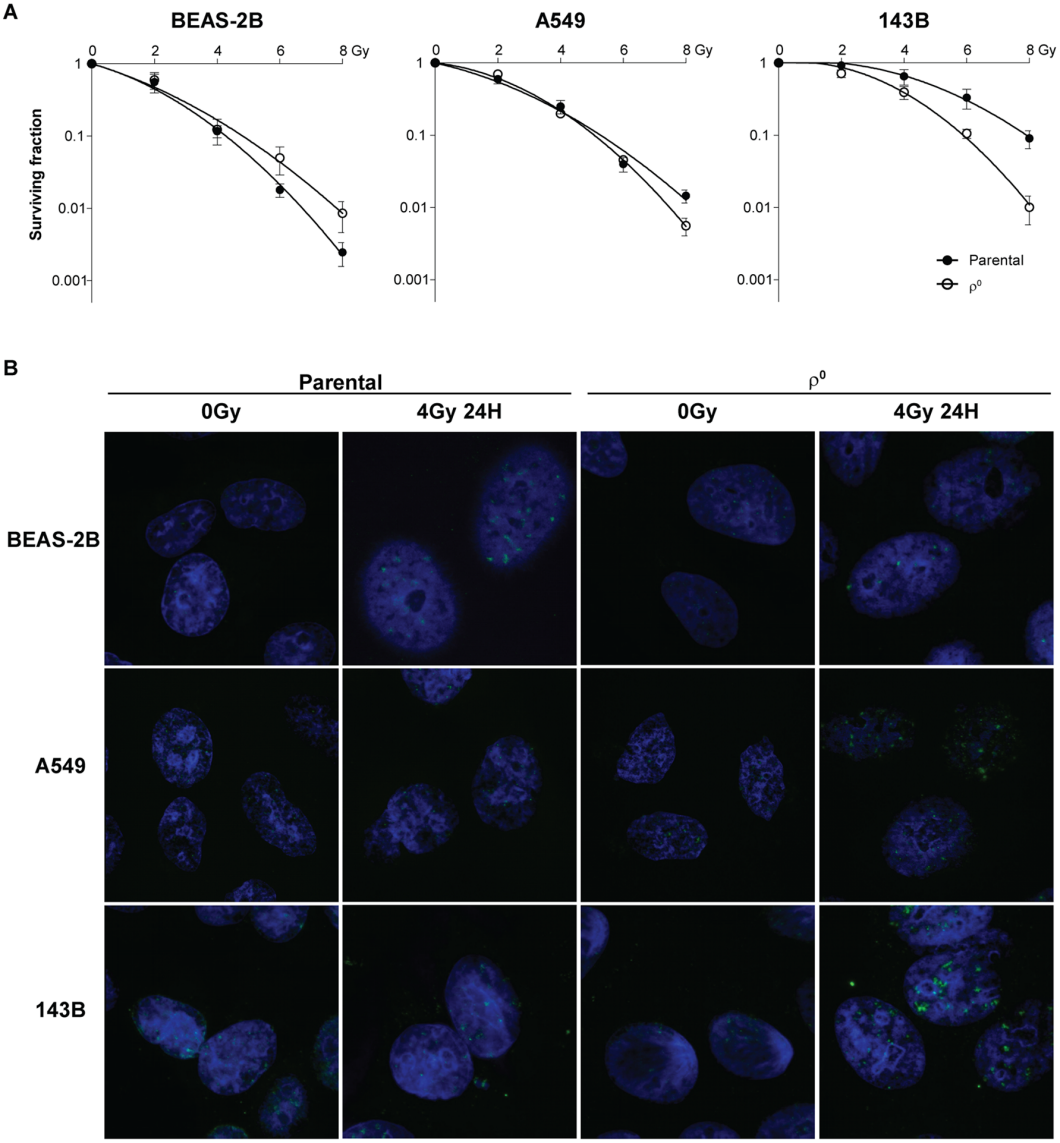
Radiation response in mtDNA depleted cell lines. **A.** Clonogenic survival plots, fitted according to the LQ model. Results show mean ± SEM from at least 3 independent biological replicates. 0 Gy conditions are sham irradiated. **B.** Representative merged fluorescent images of γH2AX foci (green) and nuclei (blue) visualizing residual foci upon irradiation.

In order to assess the influence of mtDNA depletion on radiation response, clonogenic survival assays were performed. Overall survival was increased (p<0.001) for the BEAS-2B ρ^0^ cells, while decreased for A549 ρ^0^ (p<0.01) and 143B ρ^0^ (p<0.0001) cells (Fig 2A). However, the intrinsic radiosensitivity assessed by the surviving fraction at 2Gy was not influenced (p>0.05). No diffierences in initial DNA damage were observed shortly after irradiation between mtDNA depleted and parental cells. However, changes in residual DNA damage 24 hours after 4Gy irradiation were found between both cell lines in line with the clonogenic survival data. An increased number of residual γH2AX foci was observed for the 143B ρ^0^ and to a lesser extent for the A549 ρ^0^ cells. The opposite was observed for the BEAS-2B, where the ρ^0^ cells showed a lower residual signal compared to the parental cell line (Fig 2B and S2 Fig).

**Fig 3:**
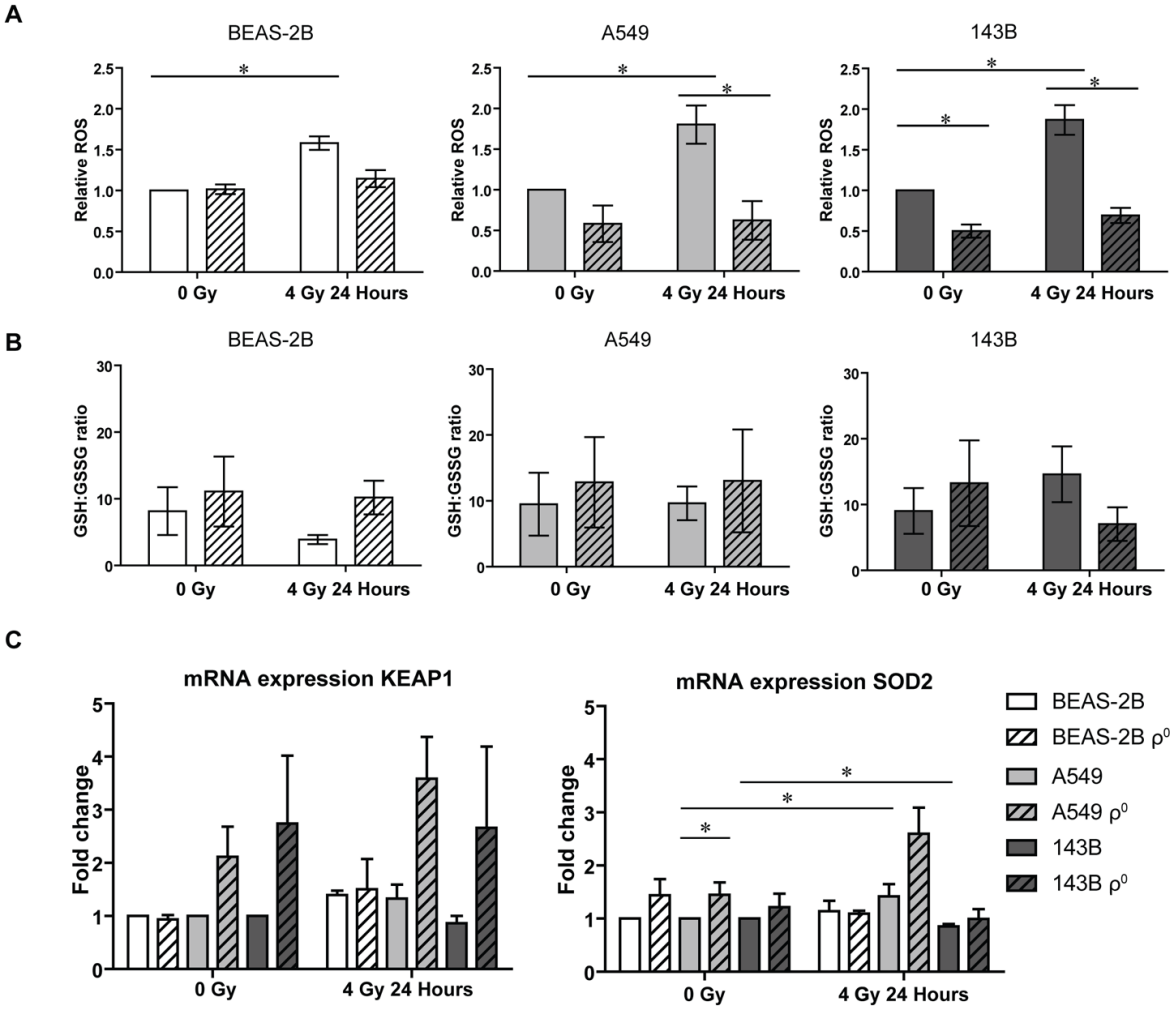
ROS and GSH levels at basal levels and 24 hours after 4Gy of irradiation. **A.** ROS levels expressed in mean intensity at baseline and 24 hours after irradiation, relative to each parental celline at baseline conditions. **B.** Ratio of GSH:GSSG levels at baseline and at 24 hours after irradiation for parental and ρ^0^ cell lines. Data represents the mean ± SEM from at least 3 independent biological repeats. mtDNA depleted cells are indicated by the dashed bars. * p<0.05. **C.** mRNA expression of KEAP1 and SOD2 24 hours after irradiation, normalized to each parental at baseline. Data represents the mean + SEM from at least 2 independent biological repeats. * p<0.05.

Radiation therapy relies on the production of reactive oxygen species (ROS) for its lethal properties. Additionally, cellular mitochondrial function plays an important role in ROS production and redox signaling. Here we assessed ROS formation (Fig 3A) at baseline (0Gy), which was found to be similar (p = 0.878) for BEAS-2B parental and ρ^0^ cell lines, while reduced for A549 (NS) and 143B (p = 0.0211) ρ^0^ cells. ROS levels were significantly increased (p<0.05) for all parental cell lines 24 hours after irradiation, while levels for the ρ^0^ cell lines remained equal. ROS formation 24 hours after irradiation was significantly lower (p<0.05) for the the A459 and 143B ρ^0^ cell lines compared to their parentals. Since “oxidative stress” indicates a disbalance between ROS and antioxidants in favor for ROS, we investigated the antioxidant capacity of the ρ^0^ cell lines using the ratio of GSH:GSSG as a proxy to assess if ROS scavenging mechanisms were altered. GSH:GSSG ratios were decreased for the BEAS-2B parental and 143B ρ^0^ cells upon irradiation. No alterations were observed for both A549 cell lines (Fig 3B). The kelch-like ECH-associated protein 1 (Keap1)—Nuclear factor erythroid 2 (NF-E2)-related factor 2 (Nrf2) pathway is an important oxidative stress response regulator and down-stream targets of Nrf2 are found to be involved in NADPH production, glutathione metabolism and utilization [23]. *KEAP1* mRNA levels were elevated for both tumor ρ^0^ cell lines at baseline and after irradiation compared to their parentals (Fig 3C). mRNA expression levels of *NRF2* were not significantly altered. Certain downstream targets of the Keap1-Nrf2 pathway such as the malic enzyme 1 (*ME1*), involved in NADPH production, displayed elevated mRNA levels in the tumor ρ^0^ cell lines (S3 Fig) while other targets such as glucose-6-phosphate dehydrogenase (*G6PD*), phosphogluconate dehydrogenase (*PGD*), NAD(P)H quinone dehydrogenase 1 (*NQO1*, involved in quinone detoxification) and glutathione peroxidase 1 (*GPX1*) did not show altered mRNA expression levels for the ρ^0^ cells (S3 Fig). Baseline levels of the antioxidant superoxide dismutase 2 (*SOD2*, *MnSOD*) were elevated in the ρ^0^ cells (Fig 3C), being significant for A549 (p = 0.0493). Irradiation increased *SOD2* expression for both parental A549 (p<0.05) and ρ^0^ (NS; p = 0.0519) cells. On the other hand, *SOD2* expression levels were decreased upon irradiation for 143B parental cells (p = 0.0093). Irradiation did not alter *SOD2* expression levels for the 143B ρ^0^ cells (p = 0.5476) neither for both BEAS-2B cell lines. Expression levels of superoxide dismutase 1 (*SOD1*, *CuZnSOD)* were not significantly altered upon irradiation for the BEAS-2B, A549 and 143B cells (S3 Fig).

## Discussion

Recently, mitochondrial DNA (mtDNA) variations have been linked to a wide variety of cancers and cancer outcome through influencing mitochondrial pathways [4] and may explain the hampered cellular bioenergetics found in many cancer types. Mitochondrial dysfunction has been found to be related to a chemoresistant phenotype *in vitro* [24,25,26,27] and chemotherapeutic agents might be associated with a decreased mitochondrial function [10,11,28]. Furthermore, several studies investigated the effect of mtDNA depletion with respect to radiotherapy by using different (tumor) cell lines with contradictory results, from a radioresistant phenotype or no difference in radiation response *in vitro* [14,15,29] to an increased radiation response *in vivo* [16].

Reactive oxygen species (ROS) are formed in the presence of oxygen and are further induced upon radiation resulting in DNA damage. Functional mitochondria are found to be essential for radiation-induced ROS production [30]. Radiation causes either cell death (mainly by mitotic catastrophy) or induces a sublethal DNA damage initiating a temporary cell cycle arrest in order to repair the damage [4]. The interplay between mitochondrial function and radiation response is of great importance in such a radiation-induced DNA damage repair [31]. As both ROS production and ATP are vital parameters for radiotherapy outcome and mitochondrial function is essential for radiation-induced DNA repair, mtDNA variations could alter the response to irradiation possibly via increased lactate production as a consequence of a reduced oxidative phosphorylation system (OXPHOS) function [26,32]. Additionally, it has been shown that Increased lactate results in a radioresistant phenotype [33]. However, in our study, no differences could be observed in cellular ATP levels for mtDNA depleted cells, in agreement with the observed compensation by glycolysis induction often found in cell lines with a dysfunctional OXPHOS system [34,35]. Although mtDNA depletion resulted in a glycolytic phenotype, no elevated lactic acid production was observed. A possible explanation for this finding could be that most lactic acid is catabolized in order to control the intracellular pH of the cell [36,37].

We hypothesized that mtDNA depletion results in a more radiosensitive phenotype related to altered ROS and ATP levels and changes in the antioxidant ROS scavaging capacity. Our data showed, in line with literature, that a varying irradiation response occured in different cell lines after mtDNA depletion. Overall, in our study the immortalized epithelial BEAS-2B ρ^0^ cell line was less radiosensitive compared to the parental line, while the tumor derived ρ^0^ cell lines (A549 and 143B) were more prone to a decreased survival upon radiation. However, the intrinsic radiosensitivity, correlated to clinical outcome after conventional fractionated radiotherapy and quantified by the survival fraction at 2Gy, was not significantly modified [38]. Residual γH2AX foci after irradiation were in agreement with the survival data, as described previously [39]. Irradiation is able to elevate ROS levels [7]. In the parental cell lines used in this study, the irradiation-induced increase in ROS levels was evident, while no increase was observed in ρ^0^ cells. Potentially, this is due to the fact that both NADH ubiquinone oxidoreductase (CI) and ubiquinol-cytochrome c oxidoreductase (CIII), the major ROS generating sites of the oxidative phosphorylation chain, might be deficient in the established ρ^0^ cells. This may explain why we found relatively lower ROS levels in the ρ^0^ tumor cell lines [40,41,42]. In contrast to the elevated ROS levels found in cells with mtDNA variations [26,43,44], Park et al. demonstrated that ρ^0^ cells can also have normal ROS levels due to the protective up-regulation of their antioxidant system during the ρ^0^ development [26].

Glutathione and SOD2 are important antioxidants that can be located in the mitochondrion [45]. Here we observed that the ratio between GSH and GSSG was decreased upon irradiation for the parental BEAS-2B and the 143B ρ^0^ cells, whearas no differences were found for the A549 cells. These data suggest that differences in radiation response between parental and mtDNA depleted cells could be determined by the antioxidant capacity of the cells. Superoxide dismutase 2 (SOD2) is an antioxidant able to scavenge superoxide radicals in the mitochondrial matrix [46] and therefore can protect cells against radiation [47]. Cells with an overexpression of SOD2 are found to have a higher cell survival [47]. We indeed observed an increase in SOD2 levels upon irradiation for the A549 parental cells. On the other hand, mitochondrial SOD2 levels where unaffected in the 143B cells. Previously, it has been reported that ρ^0^ cells originating from different tissues can have altered glutathione antioxidant mechanisms [48,49]. The Kelch-like ECH-associated protein 1- Nuclear factor erythroid 2 (NF-E2)-related factor 2 (Keap1-Nrf2) pathway is an important oxidative stress response regulator and downstream targets of Nrf2 are found to be involved in glutathione metabolism and utilization [23,50]. *KEAP1* mRNA levels were found to be elevated for both tumor mtDNA depleted lines, suggesting a role for this pathway in the antioxidant response. Some, but not all, downstream targets of the Keap1-Nrf2 pathway were found to be elevated indicating that Keap1-Nrf2 could play a role in the antioxidant capacity of ρ^0^, however this remains to be further elucidated. Furthermore, it has also been suggested that variations in culture medium can influence oxidative stress and oxidative stress responses, possibly explaining the observered differences in antioxidant mechanisms [49,51]. A well-known example is cysteine, the precursor for glutathione [51]. Therefore the use of glutathione and its effectors in culture medium should be carefully considered when elucidating the interactions of mitochondrial function and antioxidants.

Taken together, mtDNA depletion resulted in decreased proliferation for all ρ^0^ cell lines, increased clonogenic survival for the epithelial BEAS-2B cells, but reduced clonogenic survival at higher irradiation doses for the tumor cell lines. Our findings indicate that inhibition of OXPHOS might be useful to enhance radiotherapy effects, in particular for patients treated with hypofractionated radiotherapy in rapidly proliferating tumors. This is in line with the well-known OXPHOS inhibitor metformin, an U.S. food and drug administration (FDA) approved drug for diabetes leading to an improvement of tumor oxygenation and better outcome following radiotherapy [52,53]. Concluding, mtDNA depletion resulted in varying irradiation responses in different cell lines, potentially attributed to ROS and antioxidant capacity. Additionally, the influence of mtDNA variations in normal tissue and tumor tissues should be further elucidated in order to implement these novel findings in cancer treatment, both chemotherapy as well as radiation therapy.

## Supporting information

S1 Fig Representative images of the glycolysis stress test for BEAS-2B, A549 and 143B. Basal measurements are followed by subsequent injections of 10 mM glucose, 1.0 μM oligomycin and 0.1 M 2-deoxyglucose.
(TIF)

S2 Fig Quantification of representative images for γH2AX foci. γH2AX foci amount (mean ± SEM) is shown at baseline and 24 hours after irradiation, relative to each cell line at baseline conditions.
(TIF)

S3 Fig Expression of antioxidant genes. Data represents the mean ± SEM from at least 2 independent biological repeats, normalized to each parental line at baseline **p<0.01.
(TIF)

S1 Table Quantitative PCR primer sequences. (TIF)

## Abbreviations

ρ^0^: rho-zero
ATP: adenosine triphosphate
CI: NADH ubiquinone oxidoreductase
CIII: ubiquinol-cytochrome c oxidoreductase
FDA: U.S. food and drug administration
G6PD: glucose-6-phosphate dehydrogenase
GPX1: glutathione peroxidase
Gy: gray
keap1: kelch-like ECH-associated protein 1
ME1: malic enzyme 1
mtDNA: mitochondrial DNA
NQO1: NAD(P)H quinone dehydrogenase 1
Nrf2: Nuclear factor erythroid 2 (NF-E2)-related factor 2
OXPHOS: oxidative phosphorylation system
ROS: reactive oxygen species
RT: Radiotherapy
SOD: superoxide dismutase
