# Supplementary figures and images for: Distinct radiation responses after in vitro mtDNA depletion are potentially related to oxidative stress
Source: PLoS One. 2017 Aug 3;12(8):e0182508. doi: 10.1371/journal.pone.0182508 (PMC5542624; doi:10.1371/journal.pone.0182508)

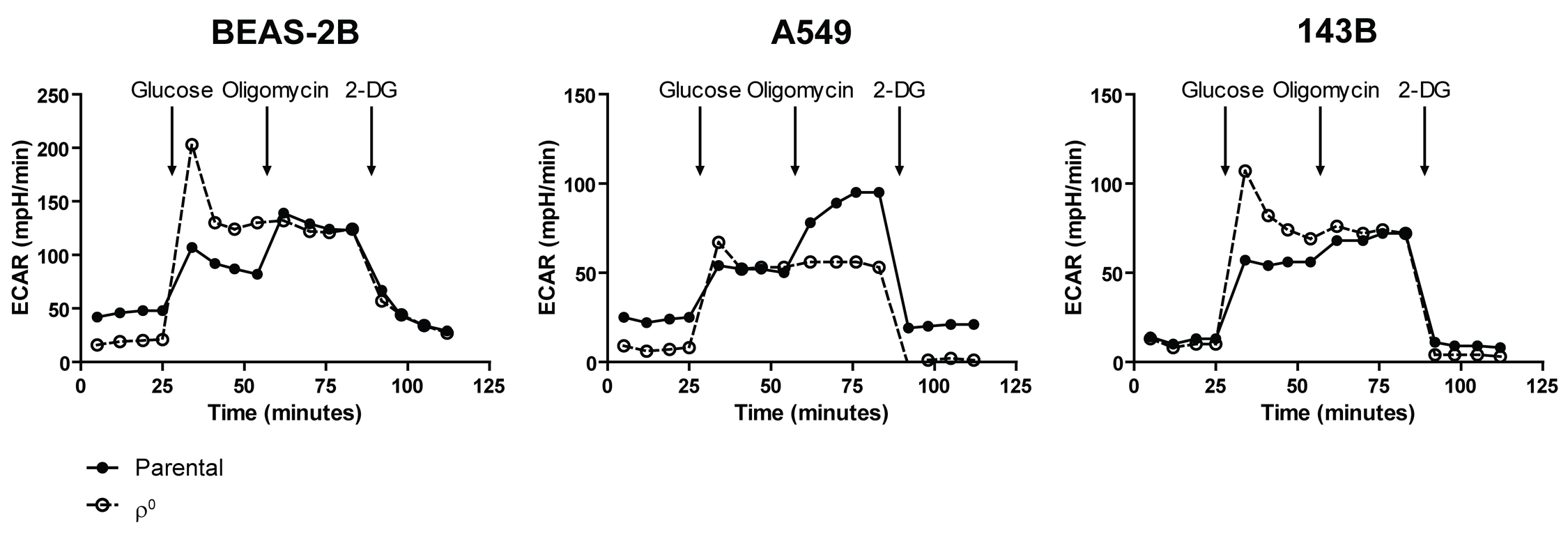

Supplement: S1 Fig — Basal measurements are followed by subsequent injections of 10 mM glucose, 1.0 μM oligomycin and 0.1 M 2-deoxyglucose. (TIF) [file pone.0182508.s001.tif]

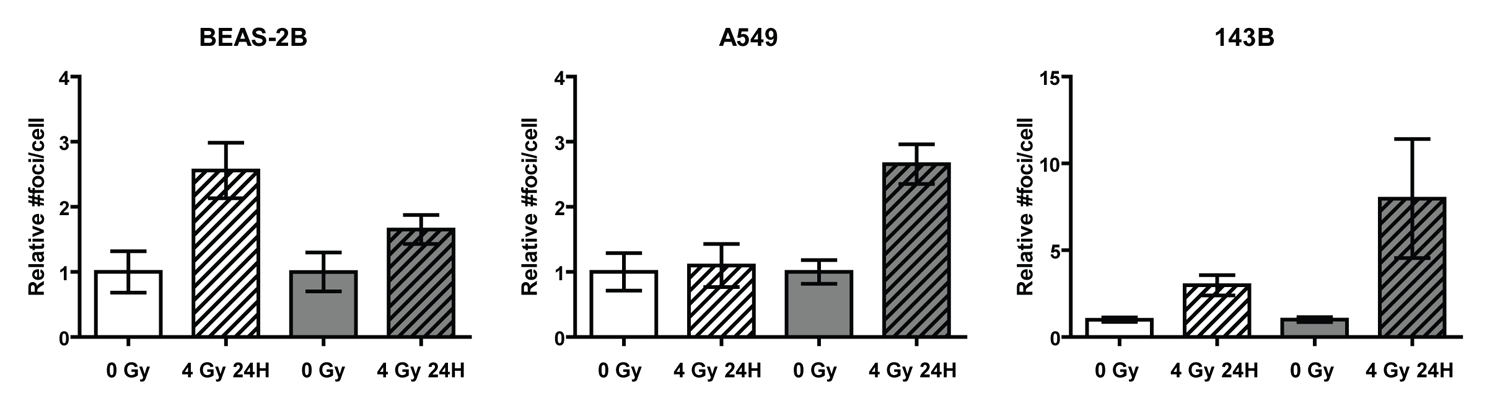

Supplement: S2 Fig — γH2AX foci amount (mean ± SEM) is shown at baseline and 24 hours after irradiation, relative to each cell line at baseline conditions. (TIF) [file pone.0182508.s002.tif]

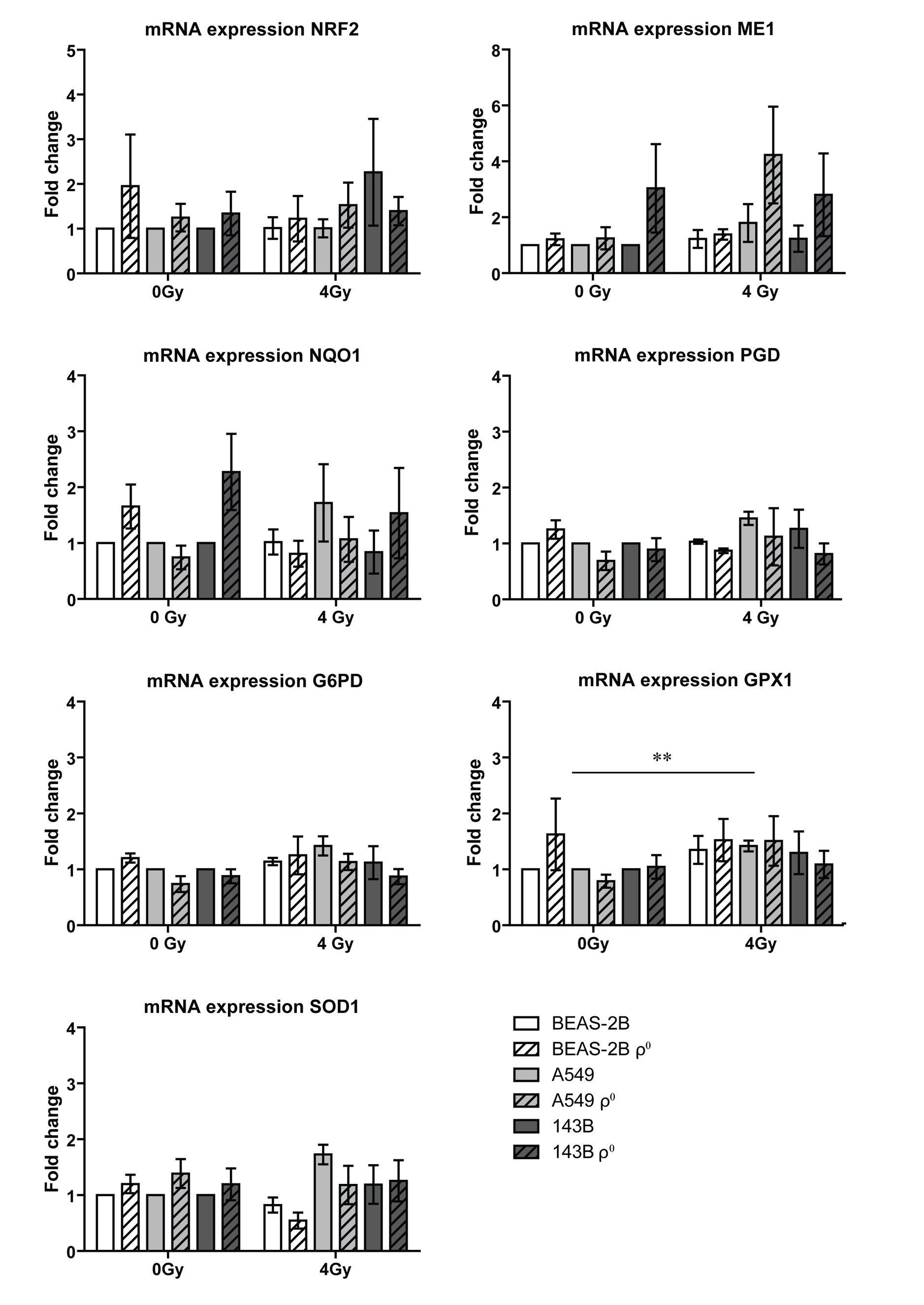

Supplement: S3 Fig — Data represents the mean ± SEM from at least 2 independent biological repeats, normalized to each parental line at baseline **p<0.01. (TIF) [file pone.0182508.s003.tif]

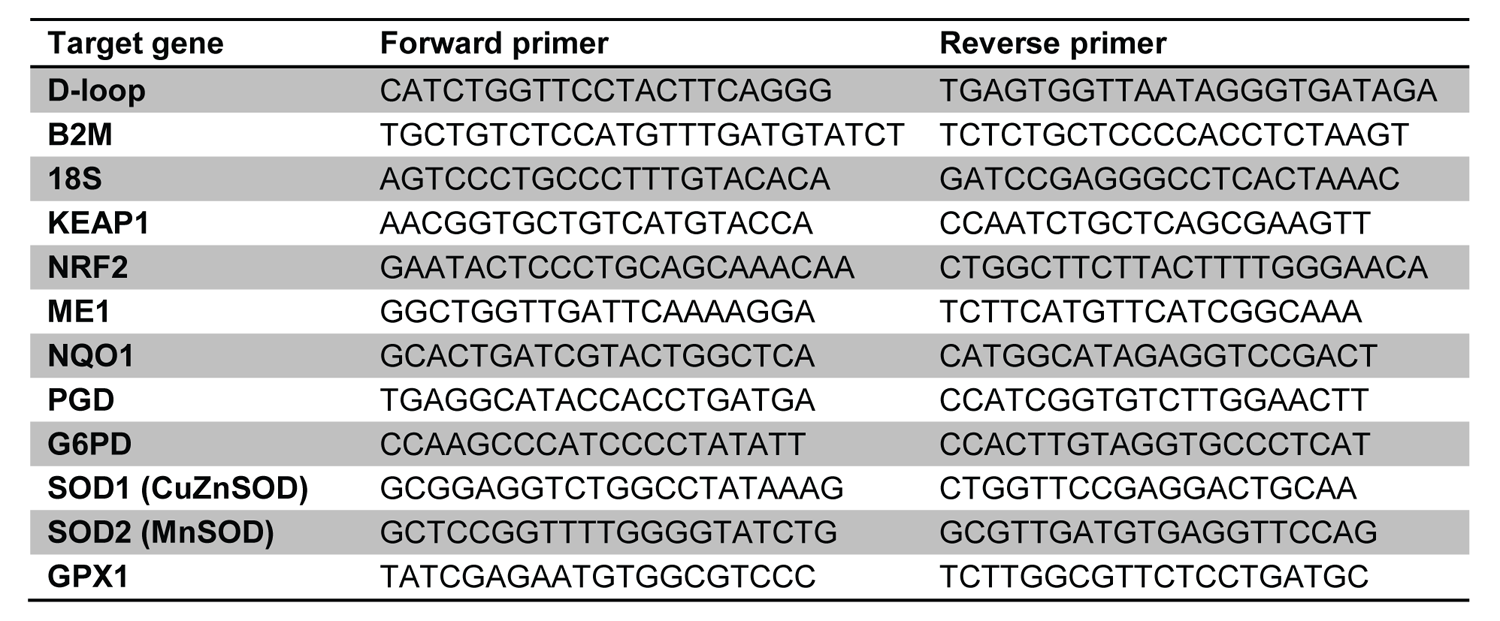

Supplement: S1 Table — (TIF) [file pone.0182508.s004.tif]
